# Editorial: Dissection of the molecular basis of fatty acid composition in oil crops and molecular breeding of oil crops with improved fatty acid composition

**DOI:** 10.3389/fpls.2022.1053735

**Published:** 2022-10-20

**Authors:** Hyun Uk Kim, Dongdong Li

**Affiliations:** ^1^Department of Bioindustry and Bioresource Engineering, Plant Engineering Research Institute, Sejong University, Seoul, South Korea; ^2^College of Tropical Crops, Hainan University, Haikou, China

**Keywords:** *arabidopsis*, *camellia*, fatty acid, oil palm, rapeseeds, rice, *physaria fendleri*, *torreya grandis*

Humans are using vegetable oil produced by various oilseed crops such as oil palm, rapeseed, and soybean for food and industry (Tokel and Erkencioglu, 2021). Vegetable oil produced from seeds and fruits of oilseed crops is in the form of triacylglycerol (TAG), and their fatty acid (FA) composition varies depending on the plant species. In the *Arabidopsis* model plant, the metabolism of FA and TAG biosynthesis has been well elucidated through genetics, molecular biochemistry, and genomics (Li-Beisson et al., 2013). However, studies on the metabolic mechanism of FA and TAG synthesis in various oilseed crops are scarce.

Given the need for research in oilseed crops, this special issue covers recent research on genetic and molecular mechanisms underlying oil-crop traits, specifically about FA. The first is the discovery of a novel transcription factor network that regulates FA synthesis and changes in FA composition by abscisic acid (ABA) treatment of oil palm. In addition, research in *Physaria fendleri* has been applied to enhance the production of industrially useful hydroxy FAs (HFAs), and transcriptomic analysis of gymnosperm *Torreya grandis* Fortune ex Lindl. kernels provided further clarity on FA metabolism. Next, important progress has been made in understanding available genetic resources through studies that assembled the chloroplast genome of the tea-oil tree *Camellia*, comprehensively mapped quantitative trait loci (QTLs) related to oil production and disease resistance in rapeseed (*Brassica napus*), and explored disease-resistance mechanisms in the lipid metabolism of rice. Finally, a rapeseed cultivar without erucic acid production in seeds was developed through gene editing. These biotechnological advances benefit the production of vegetable oils optimized for food and industrial raw materials.

## New molecular mechanisms of FA biosynthesis in oil palm, rice, and *Arabidopsis thaliana*

**Figure 1:**
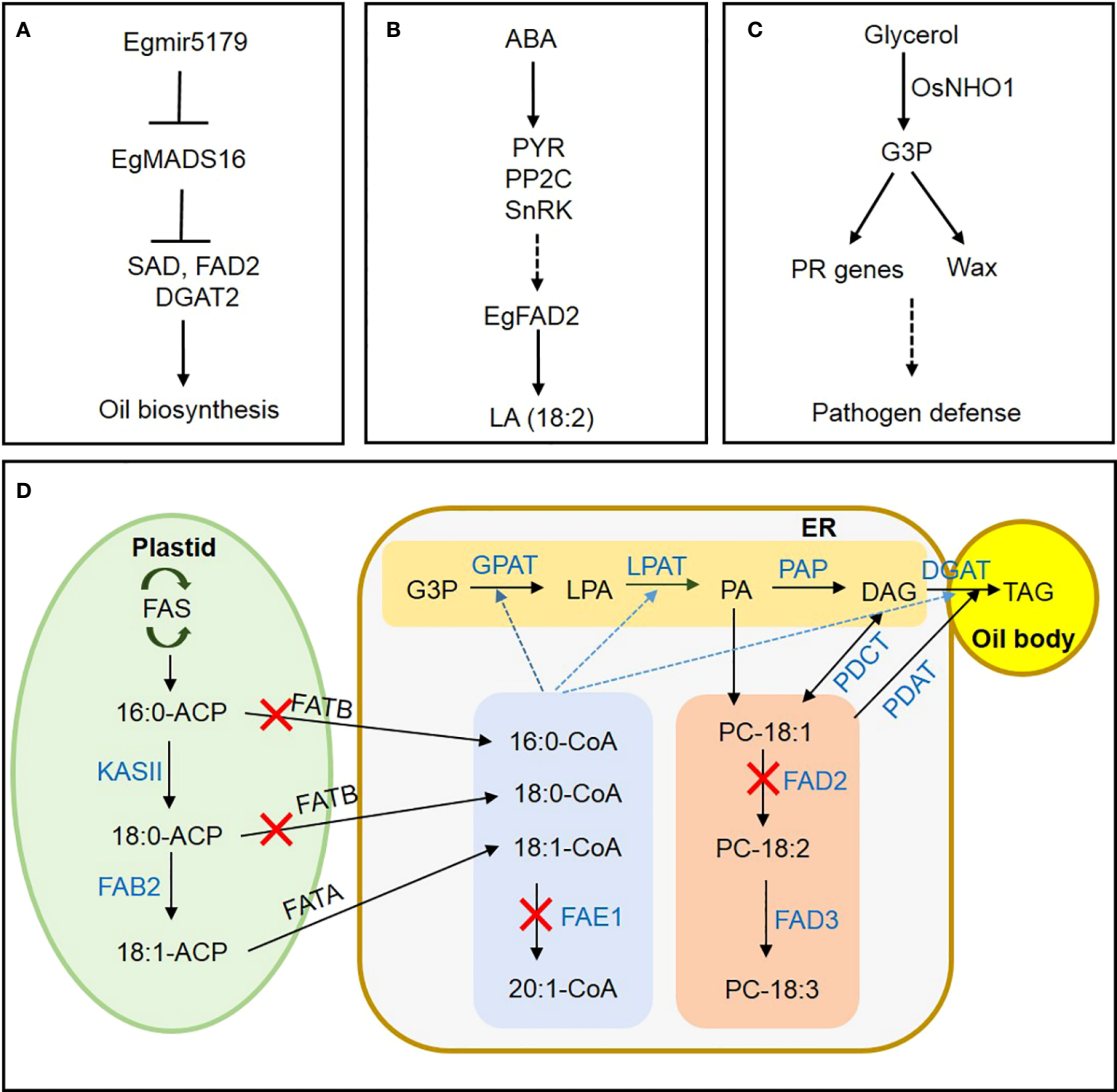
A novel mechanism for regulating lipid metabolism in oil crops and a metabolic pathway for FA and TAG synthesis in oilseeds. **(A)** In oil-palm mesocarp, mir5179 inhibits MADS16 expression and regulates the expression of oil synthesis genes *SAD*, *FAD2*, and *DGAT2*. **(B)** ABA induces linoleic acid synthesis through ABA signaling pathway in oil palm mesocarp. **(C)** Induction of disease resistance in rice through regulating PR gene expression and wax synthesis by glycerol kinase OsNHO1. **(D)** Suppression of *FAE1* expression for EA-free oil crop development. Suppressing *FATB* inhibits saturated-FA synthesis. Suppressing *FAD2* expression blocks polyunsaturated-FA synthesis, resulting in elevated production of oleic acid. Black arrows indicate the direction of FA biosynthesis and metabolism. Blue dotted arrows indicate acyl-CoA migration. Blue letters indicate enzymes involved in the metabolic pathways. Red Xs indicate enzyme inhibition. FAS, fatty acid synthase; ACP, acyl carrier protein; KAS, KASII, 3-ketoacyl-ACP synthase II; FAB2, fatty acid biosynthesis2; G3P, glycerol-3phosphate; LPA, lysophosphatidic acid; PA, phosphatidic acid; DAG, diacylglycerol; TAG, triacylglycerol; CoA, coenzyme A; GPAT, glycerol-3-phosphate acyltransferase; LPAT, lysophosphatidic acid acyltransferase; PAP, phosphatidate phosphatase; DGAT, diacylglycerol acyltransferases; PDAT, phospholipid diacylglycerol acyltransferase; PC, phosphatidylcholine; FAD2, fatty acid desaturase 2; FAD3, fatty acid desaturase 3; ER, endoplasmic reticulum; FAE1, fatty acid elongase 1; FATA, fatty acid thioesterase A; FATB, fatty acid thioesterase B; PDCT, phosphatidylcholine diacylglycerol choline phosphotransferase; PDAT, phosphatidylcholine diacylglycerol acyltransferase.

The world’s largest oil-producing crop is the oil palm (*Elaeis guineensis*), which accumulates up to 90% of its oil in the mesocarp (Bhagya et al., 2020; Zhou et al., 2020). However, few reports are available on transcription factor (TF) networks regulating lipid metabolism in oil palm (Yeap et al., 2017; Li et al., 2020). Wang et al. suggested that EgMADS16 negatively regulates *FAD2, SAD*, and *DGAT2* transcription during oil-palm mesocarp development, interacting with EgGLO1 to affect FA and triacylglycerol (TAG) biosynthesis. The authors also proposed a mechanism to explain the suppression of *EgMADS16* expression by its target *Egmir5179*, a small RNA involved in oil accumulation (**Figure 1A**). Next, a study by Shi et al. examined how ABA treatment increases the unsaturated FA linoleic acid in oil-palm mesocarp. Through transcriptome analysis, they hypothesized that exogenous ABA increases linoleic acid (18:2) accumulation *via* activating ABA signaling genes *PYR*, *PP2C*, and *SnRK*, as well as TFs such as ABI5, resulting in upregulation of *FAD2* expression (**Figure 1B**).

Glycerol kinase (GK) catalyzes the conversion of glycerol to glycerol-3-phosphate (G3P), but its physiological significance in rice defense against pathogens remains unclear. Xiao et al. confirmed that the GK gene *OsNHO1* was upregulated in *Xanthomonas oryzae* pv. *oryzae* (*Xoo*) strain PXO99. Additionally, in the transgenic rice line overexpressing *OsHNO1-OE*, GK content and *OsNHO1* transcription both increased, improving resistance to bacterial blight and blast diseases. In contrast, resistance was impaired in the OsNHO1-RNAi line. Similarly, wax content and wax-synthesis gene expression increased significantly in the overexpression line, while decreasing in the OsNHO1-RNAi line. Inhibiting OsNHO1 also led to decreased transcription of its interaction partners, OsSRC2 and OsPR. Xiao et al. concluded that OsNHO1 confers disease resistance through affecting wax content and regulating pathogenesis-related (PR) gene transcription (**Figure 1C**).

As a major seed energy source, TAG is stored in the form of fixed carbon (Baud and Lepiniec, 2009). Photosynthetic processes in chlorophyll-containing green seeds mainly involves FA synthesis by ATP and reducing agents (NADPH and NADH) produced in leaves and other green plant parts (Goffman et al., 2005; Hua et al., 2014; Zhang et al., 2016). However, the contribution of FA accumulation in non-green seeds remains uncertain. Nwafor et al. demonstrated that photosynthesis in *Arabidopsis* non-green seeds is responsible for 20% of FA synthesis, whereas photosynthesis in siliques and leaves/stems is responsible for 40%. The authors further suggested that the oxidized pentose phosphate pathway may be a source of the carbon, energy, and reducing agents required for FA synthesis during seed development.

## Genome evaluation of various oil-crop resources

Considerable effort has been devoted to developing elite rapeseed cultivars with high oil content, high yield, and disease resistance (Bao et al., 2018; Chen et al., 2018). QTLs are particularly important for deciphering agronomic traits. A recent study aligned 4,555 QTLs (identified over 25 years across 12 countries) to construct a quantitative genomic map containing 128 traits from 79 populations (Raboanatahiry et al.). These results revealed 517 regions of overlapping QTLs that harbor 2,744 candidate genes simultaneously affecting multiple traits. This data can be used to develop new rapeseed varieties.

*Torreya grandis* Fortune ex Lindl. is a commercial species of gymnosperm that produces oil-rich nuts with high unsaturated FA content in mature kernels (Shi et al., 2018). Zhang et al. compared *de novo* transcriptome and FA accumulation during kernel development of two varieties: high-oil (52.9%) *T. grandis* ‘Xifei’ and medium-oil (41.6%) *T. grandis* ‘Dielsii’. The authors inferred that differences in FA and TAG accumulation, along with related transcript expression, were the primary factors responsible for oil-content variation (oleic acid ratio, sciadonic acid ratio) between the two varieties.

*Camellia* is one of the four most commercially valued woody plants worldwide. *Camellia* oil is rich in polyphenols, saponins, and other nutritional components. Owing to its health benefits, camellia oil has strong economic competitiveness and broad market prospects (Zhu et al., 2010). Chen et al. identified an oil-tea *Camellia* species previously unknown in Hainan by comparing chloroplast genomic (cpDNA) sequences of 13 Chinese oil-tea *Camellia* samples. They concluded that cpDNA of oil-tea *Camellia* species exhibits a conserved tetrad structure with specific length polymorphisms. Additionally, simple sequence repeats (SSR) and other mutations led to an abundance of divergent hotspots in coding sequences (CDS) and intergenic space (IGS). The presence of these hotspots suggests that the entire cpDNA sequence can be used for species identification and phylogenetic analysis of *Camellia*.

## Gene editing and biotechnology to improve FA composition for human consumption and industrial uses

Brassicaceae oilseeds produce a very long-chain monounsaturated FA called erucic acid (EA, C22:1), a compound extensively used in various chemical industries (Sakhno, 2010). However, EA is not easily digested and absorbed. Moreover, high-EA rapeseed (HEAR) oil often contains glucosinolates, which have been implicated in disease. Biotechnology research is thus necessary to produce low-EA rapeseed oil (LEAR) in addition to HEAR. In their review, Wang et al. examined the EA biosynthetic pathway and EA resources in various Brassicaceae crops. The available data led them to promote commercialization of genetically modified products that improved EA content in *Brassica* oilseeds. In the same vein, Liu et al. developed rapeseed lines with significantly lower EA content by using CRISPR/Cas9 to knock out one or both *FAE1* copies in the amphidiploid plant. Knocking out *BnaC03.FAE1* decreased EA content by more than 10%, whereas knocking out both *BnaA08.FAE1* and *BnaC03.FAE1* almost completely abolished EA content. Instead, oleic acid content increased considerably. These experiments ultimately lowered seed oil content, without affecting other agricultural characteristics.

*Physaria fendleri* (Brassicaceae) accumulates the long-chain HFA lesquerolic acid (20:1OH) (Dierig et al., 2011), suggesting that this oilseed species can be an alternative crop to castors for producing industrially valuable HFAs. In support of this application, seed-specific RNAi knockdown of TAG lipase *SUGAR DEPENDENT 1* (*SDP1*) increased seed weight and total seed-oil content, without significantly affecting seedling establishment (Azeez et al.).

In their review of the biochemistry and molecular genetics underlying oil synthesis, Wallis et al. highlighted valuable tools for blocking relevant genes during seed development to lower polyunsaturated fatty acid (PUFA) content in oil. The authors introduced successful breeding programs that produced high-oleic, low-PUFA varieties of soybean, canola, and other oilseed crops. Finally, Park and Kim reviewed current case studies and future strategies for regulating FA and TAG metabolism using CRISPR/Cas9 (**Figure 1D**).

## Outlook

Vegetable oils contribute significantly to calorie intake in the human diet, but their FA composition is not ideal for nutrition nor the needs of the food industry. Therefore, the mechanisms of FA and TAG synthesis in oil crops should be identified. Clearly, to meet the growing demand for vegetable oil, further biotechnology research and better breeding programs are necessary to improve crop oil content and FA composition.

## Author contributions

All authors listed have made substantial, direct, and intellectual contributions to the work, and approved it for publication.

## Funding

This work was supported by a grant from the New Breeding Technologies Development Program (Project No. PJ016533), Rural Development Administration, Republic of Korea.

## Acknowledgments

We thank all authors, reviewers, and editorial staff who contributed to the finalization of this special issue.

## Conflict of interest

The authors declare that the research was conducted in the absence of any commercial or financial relationships that could be construed as a potential conflict of interest.

## Publisher’s note

All claims expressed in this article are solely those of the authors and do not necessarily represent those of their affiliated organizations or those of the publisher, editors, and reviewers. The publisher does not guarantee or endorse any product evaluated in this article, nor any manufacturer claims.

## References

[B1] Bao B. Chao H. Wang H. Zhao W. Zhang L. Raboanatahiry N.. (2018). Stable, environmental specific and novel QTL identification as well as genetic dissection of fatty acid metabolism in *Brassica napus* . Front. Plant Sci. 9. doi: 10.3389/fpls.2018.01018 PMC6057442 30065738

[B2] Baud S. Lepiniec L. (2009). Regulation of *de novo* fatty acid synthesis in maturing oilseeds of *Arabidopsis* . Plant Physiol. Biochem. 47, 448–455. doi: 10.1016/j.plaphy.2008.12.006 19136270

[B3] Bhagya H. P. Kalyana Babu B. Gangadharappa P. M. Naika M. B. N. Satish D. Mathur R. K. (2020). Identification of QTLs in oil palm (*Elaeis guineensis* jacq.) using SSR markers through association mapping J. Genet. 99, 19. doi: 10.1007/s12041-020-1180-4 32366730

[B4] Chen F. Zhang W. Yu K. Sun L. Gao J. Zhou X.. (2018). Unconditional and conditional QTL analyses of seed fatty acid composition in *Brassica napus* l. BMC Plant Biol. 18, 49. doi:10.1186/s12870–018-1268-7. 29566663 10.1186/s12870-018-1268-7 PMC5865336

[B5] Dierig D. A. Wang G. Mccloskey W. B. Thorp K. R. Isbell T. A. Ray D. T.. (2011). *Lesquerella*: New crop development and commercialization in the US. Ind. Crop Prod. 34, 1381–1385. doi: 10.1016/j.indcrop.2010.12.023

[B6] Goffman F. D. Alonso A. P. Schwender J. Shachar-Hill Y. Ohlrogge J. B. (2005). Light enables a very high efficiency of carbon storage in developing embryos of rapeseed. Plant Physiol. 138, 2269–2279. doi: 10.1104/pp.105.063628 16024686 PMC1183413

[B7] Hua S. Chen Z. H. Zhang Y. Yu H. Lin B. Zhang D. (2014). Chlorophyll and carbohydrate metabolism in developing silique and seed are prerequisite to seed oil content of *Brassica napus* l. Bot. Stud. 55, 34. doi: 10.1186/1999-3110-55-34 28510961 PMC5432831

[B8] Li-Beisson Y. Shorrosh B. Beisson F. Andersson M. X. Arondel V. Bates P. D.. (2013). Acyl-lipid metabolism. Arabidopsis Book 11, e0161. doi: 10.1199/tab.0161 23505340 PMC3563272

[B9] Li S. Y. Zhang Q. Jin Y. H. Zou J. X. Zheng Y. S. Li D. D. (2020). A MADS-box gene, EgMADS21, negatively regulates EgDGAT2 expression and decreases polyunsaturated fatty acid accumulation in oil palm (*Elaeis guineensis* jacq.). Plant Cell Rep. 39, 1505–1516. doi: 10.1007/s00299-020-02579-z 32804247

[B10] Sakhno L. O. (2010). Variability in the fatty acid composition of rapeseed oil: classical breeding and biotechnology. Cytol. Genet. 44, 389–397. doi: 10.3103/S009545271006010 21254621

[B11] Shi L. K. Mao J. H. Zheng L. Zhao C. W. Jin Q. Z. Wang X. G. (2018). Chemical characterization and free radical scavenging capacity of oils obtained from *Torreya grandis* fort. ex. lindl. and *torreya grandis* fort. var. *merrillii*: A comparative study using chemometrics. Ind. Crops Prod. 115, 250–260. doi: 10.1016/j.indcrop.2018.02.037

[B12] Tokel D. Erkencioglu B. N. (2021). “Production and trade of oil crops, and their contribution to the world economy,” in Oil crop genomics. Eds. Tombuloglu H. Unver T. Tombuloglu G. Hakeem K. R. (Cham: Springer). doi: 10.1007/978-3-030-70420-9_20

[B13] Von Mark V. C. and Dierig D. A. (2015). “Germplasm improvement to develop commercially viable lines of the new oilseed crop lesquerella,” in Industrial crops (New York, USA: Springer), 315–334.

[B14] Yeap W. C. Lee F. C. Shabari Shan D. K. Musa H. Appleton D. R. Kulaveerasingam H. (2017). WRI1-1, ABI5, NF-YA3 and NF-YC2 increase oil biosynthesis in coordination with hormonal signaling during fruit development in oil palm. Plant J. 91, 97–113. doi: 10.1111/tpj.13549 28370622

[B15] Zhang Y. Mulpuri S. Liu A. (2016). High light exposure on seed coat increases lipid accumulation in seeds of castor bean (*Ricinus communis* l.), a nongreen oilseed crop. Photosynth. Res. 128, 125–140. doi: 10.1007/s11120-015-0206-x 26589321

[B16] Zhou Y. Zhao W. Lai Y. Zhang B. Zhang D. (2020). Edible plant oil: Global status, health issues, and perspectives. Front. Plant Sci. 11. doi: 10.3389/fpls.2020.01315 PMC7485320 32983204

[B17] Zhu B. Zhong H. Y. Cao Q. M. Long Q. Z. (2010). Advance in research on bioactive compounds in camellia spp. Nonw. For. Res. 28, 140–145. doi: 10.14067/j.cnki.1003-8981.2010.03.026

